# Age- and sex-specific differences in blood-borne microvesicles from apparently healthy humans

**DOI:** 10.1186/s13293-015-0028-8

**Published:** 2015-05-11

**Authors:** Callie M Gustafson, Alex J Shepherd, Virginia M Miller, Muthuvel Jayachandran

**Affiliations:** Department of Surgery, Mayo Clinic, 200 First St. SW, Rochester, MN 55905 USA; Physiology and Biomedical Engineering, Mayo Clinic, 200 First St. SW, Rochester, MN 55905 USA

**Keywords:** Anticoagulant vesicles, Extracellular vesicles, Flow cytometry, Microparticles, Procoagulant vesicles, Sex differences

## Abstract

**Background:**

Sex differences in incidence of cardiovascular disease may reflect age-associated intravascular cellular activation resulting in shedding of cell membrane-derived bioactive microvesicles (MV or microparticles) into the blood. Concentrations of cell-specific MV in blood have the potential to be a diagnostic/prognostic marker of pathology, but ranges of MV must first be established in healthy individuals. This study identified cellular origin of blood-borne MV >0.2 μm in blood of apparently healthy women and men aged from 20–70 years.

**Methods:**

Venous blood from apparently healthy participants in the Mayo Clinic Biobank was collected into tubes containing protease inhibitors as the anticoagulant. MV were isolated by standardized differential centrifugation and characterized by digital flow cytometer. Each cellular origin of MV was verified by two different antibodies with strong correlation between the two distinct antibodies (e.g., for platelet-derived MV, *r*^2^ = 0.97).

**Results:**

MV derived from platelets were the most abundant type of MV in blood from women and men in all age groups. Total numbers of phosphatidylserine, P-selectin, and platelet- and endothelium-derived MV were significantly (*P* < 0.05) greater in women than men. Numbers of MV from erythrocytes and stem/progenitor cells were significantly lower in premenopausal women than age-matched men. Number of tissue factor pathway inhibitor positive MV were significantly (*P* < 0.05) lower whereas erythrocyte-derived MV were significantly higher in postmenopausal women compared to premenopausal women. In women, there was a positive relationship between age and erythrocyte-derived MV (*ρ* = 0.28; *P* = 0.009), while in men adipocyte-derived MV increased with age (*ρ* = 0.33; *P* = 0.01).

**Conclusions:**

This study provides ranges for cellular origin of blood-borne MV in age-matched, apparently healthy women and men from which to compare diagnostic and prognostic uses of blood-borne MV in larger studies and patient population. In addition, sex- and age-specific differences in phosphatidylserine, platelet-, endothelium-, erythrocyte-, and adipocyte-derived blood-borne MV may contribute to differential progression of cardiovascular disease in women compared to men.

## Background

Microvesicles (MV or microparticles) are submicron-size sealed vesicles shed from the plasma membrane of activated cells. Blood-borne MV participate in cell-cell communication, in the transfer of bioactive molecules between cells, and in procoagulant, anticoagulant, and proinflammatory functions, thus, implicating their contribution to pathophysiology of vascular disorders [1-8]. Cell surface proteins/receptors that come from the parent cell are a part of the MV membrane and can be used to signal other cells. Most MV originating from activated cells carry surface phosphatidylserine, a type of negatively charged phospholipid that acts as a catalytic site for the generation of thrombin that is required for the conversion of fibrinogen to fibrin clots [9,10]. MV originating from activated platelets, leukocytes, erythrocytes, and endothelial cells have been linked to cardiovascular and thrombotic complications and other diseases [4,7,10,11].

The incidence of cardiovascular disease increases with age and differs between men and women [12]. Changes in endogenous circulating concentrations of sex hormones such as testosterone, progesterone, and estrogen may modulate the risk for cardiovascular disease in men and women through direct effects on the vascular endothelium and smooth muscle and indirectly through modulation of blood elements and synthesis of inflammatory and coagulation proteins [13-21]. Procoagulant activity of the blood can be monitored by identification of specific MV, the number of which is modulated in part by estrogen [22]. One study in healthy men (age not mentioned) identified the most abundant MV in the blood originated from platelets followed by endothelial cells, granulocytes, and erythrocytes [9]. Similar results were observed in healthy menopausal women (45–56 years old) [22], but similar studies by decade of life for healthy women and men are lacking.

The cellular origin and characteristics of blood-borne MV have been studied in several types of cardiovascular and inflammatory diseases and in some types of cancer [6,23-28]. These studies suggest that MV could be used as specific candidate markers for diagnosis and prognosis of early and late disease processes. However, standard reference ranges for the type and number of MV present in healthy individuals based on their sex, and age has yet to be established. Thus, the aim of this study was to characterize and quantify the type and number of larger (higher than 0.2 μm, a lowest detection limit of digital flow cytometer) MV present in the blood of apparently healthy women and men in the age range of 20–70 years old by digital flow cytometer using standardized methodology [10,29]. It was hypothesized that the type of MV shifts with age from ones that originate from platelets to those of more procoagulant or thrombogenic origins such as leukocytes and vascular endothelium. This study also tested whether or not men have more thrombogenic MV compared to premenopausal women of the same age, but have similar thrombogenicity to age-matched postmenopausal women.

## Methods

### Antibodies and other reagents

A detailed summary of proteins and antibodies used in this study for identification and quantification of blood-borne MV >0.2 μm by digital flow cytometer is presented in Table 1. Fluorescein isothiocyanate (FITC) or R-phycoerythrin (PE)-conjugated rat anti-mouse IgG and mouse anti-rabbit IgG isotype control antibodies were purchased from BD Biosciences, San Jose, CA, USA and Santa Cruz Biotechnology, Inc., Dallas, TX, USA, respectively. FITC and/or PE-conjugated recombinant annexin-V, mouse anti-human cluster differentiation 2 (CD2), CD3, CD11c, CD14, CD19, CD34, CD41, CD42a, CD45, CD54 (ICAM-1), CD62P, CD62E, CD68, CD70, CD86, CD106 (VCAM-1), CD144, CD146, CD235a, and p16 set antibodies and TruCOUNT™ (4.2 μm) beads were purchased from BD Biosciences, San Jose, CA, USA. Fluorescent latex beads (1 and 2 μm) were purchased from Sigma-Aldrich, St. Louis, MO, USA. Fluoresbrite® microparticles (0.2, 0.5, 1, and 2 μm) were purchased from Polysciences, Inc., Warrington, PA, USA. PE-conjugated anti-human glycophorin C (CD236) antibody was purchased from Novus Biologicals, LLC, Littleton, CO, USA. FITC-conjugated mouse anti-human tissue factor antibody was purchased from American Sekisui Diagnostics, LLC, Stamford, CT, USA. FITC-conjugated rabbit anti-human tissue factor pathway inhibitor and rabbit anti-FUCA1 (alpha-L-fucosidase) antibodies were purchased from Bioss Inc., Woburn, MA, USA. PE- or FITC-conjugated rabbit anti-human fatty acid binding protein 4 (FABP4), anti-preadipocyte factor-1 (Pref-1), and mouse anti-human C-kit/CD117 antibodies were purchased from LifeSpan BioSciences, Inc., Seattle, WA, USA. 4-(2-Hydroxyethyl)-1-piperazineethanesulfonic acid (HEPES) and Hanks’ balanced salts were purchased from Sigma Chemicals Co., St. Louis, MO, USA. All other reagents and solvents used in this study were of analytical/reagent grade.

**Table 1:** **Fluorophore conjugated proteins/antibodies used to characterize blood-borne microvesicles by flow cytometry**

**Parameters**	**Protein/antibodies used for characterization**	
Procoagulant (or) thrombogenic microvesicles		
Phosphatidylserine positive	Recombinant annexin-V	
Tissue factor positive	Tissue factor	
P-selectin positive	P-selectin	
Anticoagulant microvesicles		
Tissue factor pathway inhibitor (TFPI) positive	TFPI	
Cellular adhesion molecules positive microvesicles		
Inter cellular adhesion molecule-1 (ICAM-1) positive	ICAM-1	
Vascular cell adhesion molecule-1(VCAM-1) positive	VCAM-1	
Cellular origin of microvesicles	Marker 1: protein/antibodies	Marker 2: protein/antibodies
Erythrocyte-derived	CD235a	CD236
Leukocyte-derived	CD45	CD11c
Monocyte-derived	CD14	CD86/CD68
T-cell-derived	CD3	CD2/CD69
B-cell-derived	CD19	CD70
Platelet-derived	CD42a	CD41
Endothelium-derived	CD62E	CD146/CD144
Adipocyte-derived	Fatty-acid-binding protein 4 (FABP4)	Pre-adipocyte factor-1 (Pref-1)
Stem/progenitor cell-derived	CD117	CD34
Senescent cell-derived	p16-set	Alpha-L-fucosidase

### Study participants

This study was approved by the Institutional Review Board at Mayo Clinic, Rochester, MN. Participants in this study were women (*n* = 82) and men (*n* = 62) aged 20–70 years who volunteered to be part of the Mayo Clinic Individualized Medicine Biobank. The inclusion and exclusion criteria used to obtain samples from the Individualized Medicine Biobank were based on those used for the Kronos Early Estrogen Prevention Study and for what might be considered ‘apparently healthy’ persons who volunteered for the Biobank Study [30,31]. Women were divided into two groups based on self-reported menstrual status (premenopausal and postmenopausal). No woman less than 50 years old reported being postmenopausal. Men were matched by age with women in each group. Exclusion criteria included self-reported (for women) known BRCA mutation positive genotype, complex endometrial disease, endometrial cancer, hysterectomy, use of oral contraceptives, and use of selective estrogen receptor modulators (SERMs) such as Raloxifene, Tamoxifen. Exclusion for both women and men were *in utero* exposure to diethylstilbestrol (DES; maternal treatment); current smoking more than ten cigarettes/day; body mass index >35 (kg/m^2^); history of clinical cardiovascular disease including myocardial infarction, angina, or congestive heart failure; history of cerebrovascular disease including stroke or transient ischemic attack; history of thromboembolic disease (deep vein thrombosis or pulmonary embolus); history of untreated (no cholecystectomy) gallbladder disease; dyslipidemia (LDL cholesterol >190 mg/dL); current or recent (3 months) use of lipid-lowering medications or supplements (e.g., statin, fibrate, >500 mg/day of niacin, red rice yeast); nut allergy; uncontrolled hypertension (systolic BP >150 and/or diastolic BP >95); and history of, or prevalent, chronic diseases including any cancer (other than basal cell skin cancers), renal failure, cirrhosis, diabetes mellitus, and endocrinopathies other than adequately treated thyroid disease, known HIV infection and/or medications for HIV infection, active severe clinical depression, and dementia.

### Blood sample collection

Venous blood was collected into protease inhibitors (1 μM hirudin to inhibit thrombin plus 10 μM soybean trypsin to inhibit factor Xa) to prepare platelet-free plasma by double centrifugation at 3,000 *g* for 15 min within 30 min of blood collection [29]; aliquots of platelet-free plasma were frozen at −70°C until MV analysis. Freeze and thaw of plasma do not affect the concentration of microvesicles [29]. Serum was not collected in this study, and sex hormones were not measured.

### Blood-borne MV isolation, identification, and characterization by flow cytometry

The detailed method for the isolation, identification, separation, and quantification of blood-borne MV is published by our group [10,22,29,32]. Briefly, plasma was separated from whole blood by double centrifugation at 3,000 *g* for 15 min. Contamination of the plasma by platelets and other cells was monitored by Coulter counter and flow cytometry. After validation, this plasma sample was centrifuged at 20,000 *g* for 30 min for MV isolation [29]. The pellets of MV were washed and reconstituted with twice filtered (0.2 μm pore membrane filter) 20 mM Hepes/Hank’s buffer (pH 7.4) and then vortexed for 1–2 min before staining with antibodies. For identification, digital flow cytometer (FACSCanto™, BD Biosciences, San Jose, CA, USA) was used to define MV by size calibration beads and positive annexin-V-fluorescence [29]. Gates to define size are set using an internal standard of 0.2, 0.5, 1, and 2 μm latex or silicon beads [29]. The lowest detection limit for the digital flow cytometer based on size calibration beads is 0.2 μm [10,29]; therefore, MV detection was set at this limit. For quantification, samples included a known quantity of beads (TruCOUNT™, BD Biosciences, San Jose, CA, USA) of 4.2 μm diameter.

**Figure 1:**
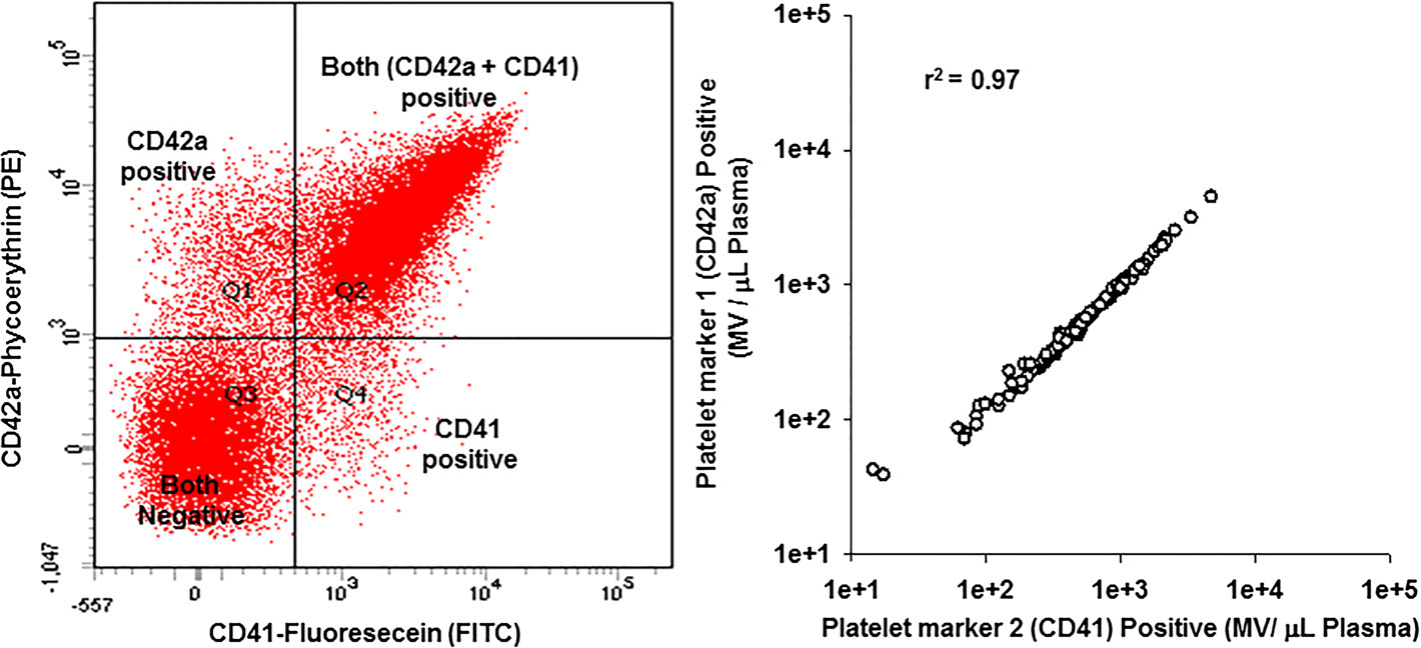
Typical fluorescence dot plot and correlation of two distinct antibodies for platelet-specific antigens. Left, typical fluorescence dot plot (Q; quadrant) from the MV gate of the light scatter by FACSCanto™ flow cytometer showing the spectra for CD41and CD42a positive MV of activated platelets from apparently healthy women and men. All (100%) platelet-derived MV did not carry both CD41and CD42a antigens (Q2). Right, correlation of two distinct antibodies for platelet-specific antigens [CD41 (Q4+Q2) and CD42a (Q1+Q2)] binding to MV isolated from the same blood sample of apparently healthy women and men. A similar pattern was observed using two distinct antibodies for other cell-specific identification of the MV (data not shown).

All antibodies were directly conjugated with either fluorescein (FITC) or PE. Cellular origins of blood-borne MV were verified using two different fluorophores (FITC and PE) conjugated to two distinct cell surface marker antibodies considered to be specific for each cell type (Table 1). The FITC- and PE-conjugated rat anti-mouse IgG and mouse anti-rabbit IgG isotype control antibodies were used as controls and for threshold setting for fluorescence dot or scatter plot [29,33]. MV were separated by fluorescence scatter or dot plot quadrants (Q) derived MV gate of light scatter plot in the presence PE (Q1+Q2) and FITC (Q4+Q2) or absence of both (Q3) of staining (Figure 1). The absolute numbers of fluorophores positive MV was calculated based on counts of calibration beads. The absolute count of specific fluorophore positive MV = number of counts in each fluorophore positive MV region/number of counts in TruCOUNT™ bead region × number of beads per test (spiked known count)/test volume [29]. The same calculation applied to quantitation of MV positive or negative for annexin-V and each cell membrane-specific antibody. Numbers of heterogeneous size of isolated blood-borne MV from 0.2–1 μm are reported in this study.

### Statistical analysis

Analyses of MV data from the flow cytometry were performed using the software JMP from the Statistical Analysis System (S.A.S.), Cary, NC, USA. The distribution of blood-borne MV (normal or skewed) depends on the cell of origin. Data from each marker positive blood-borne MV are presented as median with 25th and 75th percentiles. Differences in concentration of MV between women and men were analyzed by Wilcoxon/Kruskal-Wallis test (rank sum test) or Student’s *t*-test using JMP or Sigma plot software with significance accepted at *P* < 0.05. Nonparametric Spearman’s rank correlation coefficient test was performed to determine the association between MV identified by two different cell surface-specific markers and to determine age-associated changes in blood-borne MV numbers.

## Results

### Sex differences in populations of procoagulant (thrombogenic), anticoagulant, and cellular adhesion molecules positive MV

Phosphatidylserine (procoagulant) positive MV were the most abundant type of MV in the blood of both women and men of all age groups (Table 2). Other procoagulant (tissue factor and P-selectin)MV were not as abundant as the phosphatidylserine positive MV (Table 2). Numbers of phosphatidylserine and P-selectin positive procoagulant MV, but not tissue factor positive MV, were significantly (*P* < 0.05) higher in women compared to age-matched men (Table 2). There were no statistically significant differences in the numbers of MV positive for anticoagulant (tissue factor pathway inhibitor ) and cellular adhesion molecules (ICAM-1 and VCAM-1) between age-matched women and men (Table 2). The ratio of tissue factor (procoagulant)/tissue factor pathway inhibitor (anticoagulant) positive MV did not differ between women (median, 1.8 (1, 4, 25th, and 75th percentile, respectively)) and men (median, 1.7 (1, 6, 25th, and 75th percentile, respectively)).

**Table 2:** **Procoagulant, anticoagulant, and cellular adhesion molecules positive microvesicles from apparently healthy women and men separated by age, sex, and menopause status**

**Blood-borne microvesicles (MV)**	**20–70 years**		**20–65 years**		**51–70 years**	
	**Women (** ***N*** **= 82)**	**Men (** ***N*** **= 62)**	**Premenopausal women (** ***N*** **= 58)**	**Men (** ***N*** **= 59)**	**Postmenopausal women (** ***N*** **= 24)**	**Men (** ***N*** **= 33)**
Procoagulant positive MV/μL plasma						
Phosphatidylserine MV	921* (439, 1,443)	660 (466, 1,002)	827* (354, 1,406)	652 (463, 1,002)	979 (528, 1,555)	692 (543, 942)
Tissue factor MV	17 (7, 29)	11 (5, 26)	16 (8, 29)	10 (5, 25)	21 (6, 31)	17 (4, 27)
P-selectin MV	85* (45, 202)	62 (32, 114)	90* (31, 202)	61 (32, 113)	82 (49, 302)	61 (18, 111)
Anticoagulant positive MV/μL plasma						
Tissue factor pathway inhibitor MV	7 (4, 20)	6 (2, 17)	9 (4, 21)	7 (2, 18)	5^†^ (3, 17)	8 (3, 19)
Cellular adhesion molecules positive MV/μL plasma						
ICAM-1 MV	14 (7, 31)	14 (7, 29)	15 (3, 28)	13 (7, 29)	20 (9, 39)	19 (6, 31)
VCAM-1 MV	6 (3, 20)	7 (2, 16)	7 (3, 21)	7 (2, 17)	6 (4, 14)	8 (4, 21)

Data are presented as median with 25th and 75th percentiles.

*Statistically significant (*P* < 0.05) differences in concentration of MV between women and age-matched men. ^†^Statistically significant (*P* < 0.05) differences in concentration of MV between postmenopausal women and premenopausal women.

A comparison of premenopausal women with age-matched men showed that phosphatidylserine and P-selectin positive MV were higher in women whereas there were no statistically significant differences in tissue factor, tissue factor pathway inhibitor, and cellular adhesion molecules (ICAM-1 and VCAM-1) positive MV population (Table 2). There were no statistically significant sex differences in procoagulant, anticoagulant, and cellular adhesion molecules positive MV between postmenopausal women and age-matched men (Table 2).

### Validation of cellular origin of blood-borne MV using two distinct cell surface marker antibodies by digital flow cytometer

Typical fluorescence spectra showing the separation of the scatter plot from the FACSCanto™ flow cytometric analysis of isolated MV stained with two different platelet surface marker antibodies (CD41 and CD42a) are presented in Figure 1. In most cases, such as with antibodies CD41 and CD42a, both antibodies are found almost exclusively on platelet-derived MV, but not all (100%) platelet-derived MV carry both markers (Q2; quadrant 2) calculated from fluorescence dot plots (Figure 1). The yield of isolated platelet-derived MV from the same sample of platelet-free plasma was equal when calculated separately for each marker alone (e.g., FITC-conjugated CD41 (Q4+Q2) positive and PE-conjugated CD42a (Q1+Q2) positive (*r*^2^ = 0.97, Figure 1). Similar results were obtained with two different cell membrane-specific markers conjugated with either FITC or PE for determining the cellular origins of other blood-borne MV (data not shown).

### Sex differences in cellular origin of blood-borne MV

The absolute counts of MV positive for a cell-specific (two distinct) marker were the same for the given cell type, therefore, results are presented only from one marker of each cell-specific MV in Table 3. Only numbers of MV positive for markers for platelets and vascular endothelium differed significantly (*P* < 0.05) between women and men when all ages were combined (Table 3). However, when evaluated relative to menopausal status, endothelium-derived MV were higher in premenopausal women compared to age-matched men; whereas, erythrocyte- and stem/progenitor cells-derived MV were lower (Table 3). Numbers of endothelium-derived MV were higher in postmenopausal women compared to age-matched men (Table 3).

**Table 3:** **Cell-derived blood-borne microvesicles from apparently healthy women and men separated by age, sex, and menopausal status**

**Blood-borne microvesicles**	**20–70 years old**		**20–65 years old**		**51–70 years old**	
	**Women (** ***N*** **= 82)**	**Men (** ***N*** **= 62)**	**Premenopausal women (** ***N*** **= 58)**	**Men (** ***N*** **= 59)**	**Postmenopausal women (** ***N*** **= 24)**	**Men (** ***N*** **= 33)**
Cellular origin of MV/μL plasma						
Total erythrocyte-(CD235a) derived MV	57 (23, 107)	73 (44, 124)	49* (19, 96)	76 (45, 142)	77^†^ (50, 177)	69 (40, 165)
Total leukocyte (CD45)-derived MV	23 (16, 35)	23 (17, 38)	23 (16, 38)	23 (17, 39)	28 (16, 35)	25 (18, 41)
Monocyte (CD14)-derived MV	12 (7, 27)	11 (6, 26)	12 (7, 27)	11 (6, 25)	12 (6, 27)	16 (8, 38)
T-cell (CD3)-derived MV	6 (4, 9)	5 (3, 9)	6 (4, 9)	5 (3, 10)	6 (4, 13)	5 (3, 7)
B-cell (CD19)-derived MV	10 (5, 16)	7 (4, 17)	8 (4, 15)	7 (4, 17)	11 (6, 23)	7 (4, 19)
Platelet (CD42a)-derived MV	626* (293, 1156)	486 (342, 868)	687 (261, 1126)	492 (349, 853)	529 (362, 1298)	508 (364, 921)
Endothelium (CD62E)-derived MV	17* (9, 30)	10 (4, 22)	16^*^ (7, 24)	10 (5, 22)	20^*^ (11, 41)	9 (5, 21)
Adipocyte (FABP4)-derived MV	5 (3, 15)	5 (2, 10)	5 (3, 14)	5 (1, 10)	5 (2, 18)	6 (3, 14)
Stem/progenitor cell (CD117)-derived MV	4 (2, 8)	6 (3, 10)	3* (2, 7)	6 (3, 10)	5 (3, 8)	7 (5, 10)
Senescent cell (p16-set)-derived MV	7 (3, 13)	5 (2, 12)	7 (3, 14)	5 (2, 12)	6 (2, 12)	6 (2, 18)

Data are presented as median with 25th and 75th percentiles.

*Statistically significant (*P* < 0.05) differences in concentration of MV between women and age-matched men. ^†^Statistically significant (*P* < 0.05) differences in concentration of MV between postmenopausal women and premenopausal women.

### Differences in blood-borne MV between premenopausal and postmenopausal women

Number of tissue factor pathway inhibitor positive MV were significantly lower (*P* < 0.05, Table 2); whereas, numbers of erythrocyte-derived MV were significantly higher in postmenopausal women compared to premenopausal women (Table 3).

### Age-associated changes in numbers of blood-borne MV in women and men

**Figure 2:**
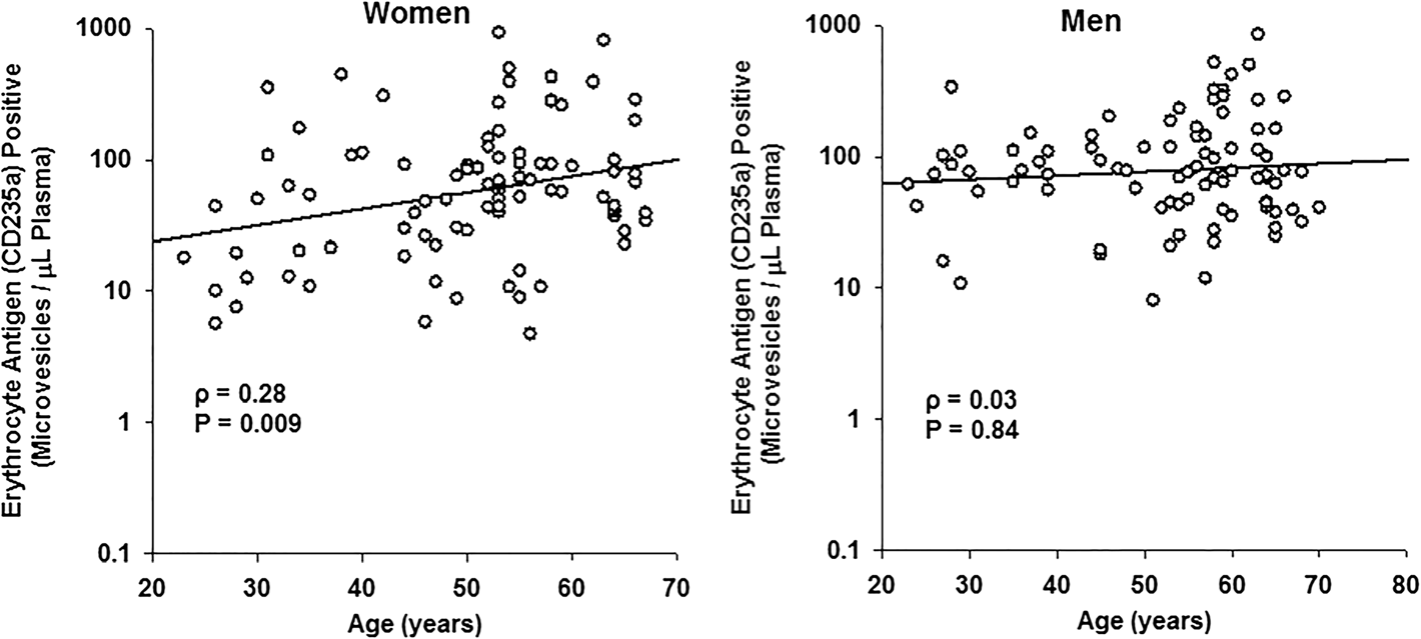
Sex differences in age-associated changes in erythrocyte-derived MV from apparently healthy women and men. Erythrocyte-derived MV show a positive correlation with age in women, whereas no correlation for erythrocyte-derived MV with age is observed in men.

**Figure 3:**
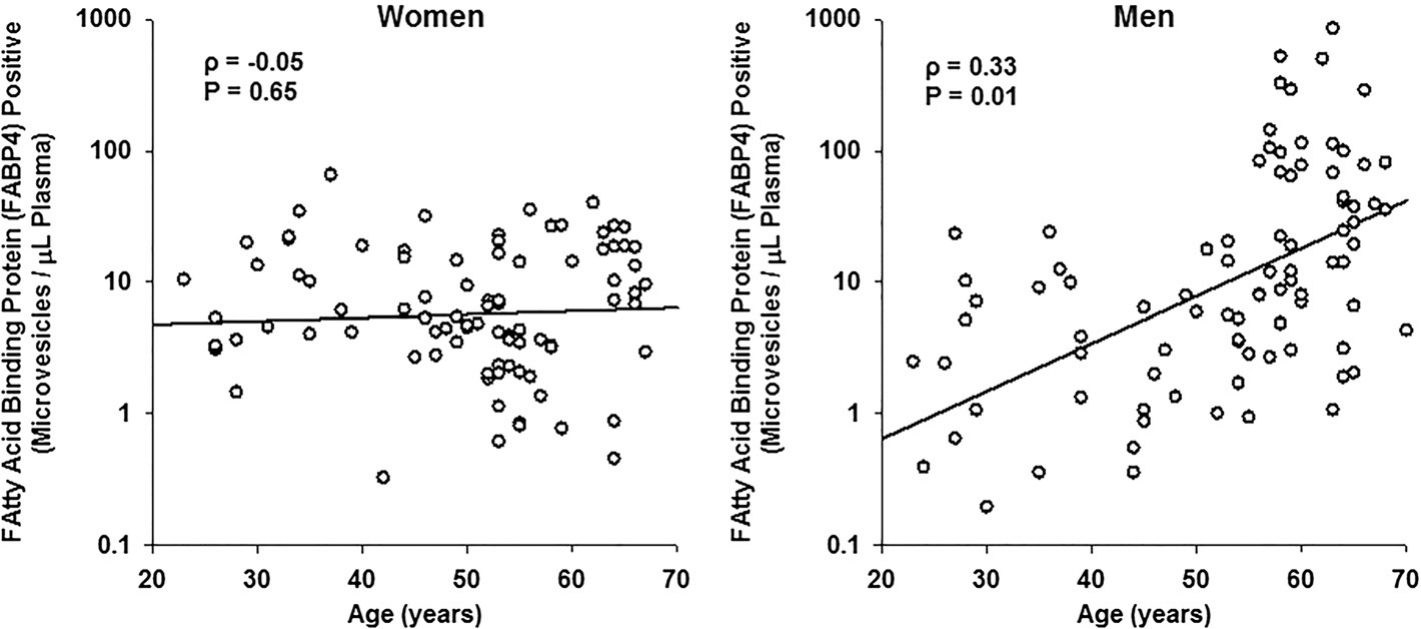
Sex differences in age-associated changes in adipocyte-derived MV from apparently healthy women and men. No correlation of adipocyte-derived MV with age was observed in women; whereas in men, positive correlation was observed between adipocyte-derived MV and age.

Numbers of erythrocytes-derived MV correlated (*ρ* = 0.28; *P* < 0.009) positively with age in women but not in men (Figure 2) whereas numbers of adipocyte-derived MV correlated (*ρ* = 0.33; *P* < 0.01) positively with age in men but not in women (Figure 3). There was a nominal negative correlation between tissue factor pathway inhibitor with age in women (*ρ* = −0.22; *P* = 0.049) but not in men (*ρ* = −0.04; *P* = 0.731). No correlations were observed between numbers of other types of blood-borne MV and age in either women or men (data not shown).

## Discussion

This study provides the ranges of MV positive for molecules involved in procoagulation, anticoagulation, cellular adhesion, and the cellular origin of MV in apparently healthy women and men between the ages of 20–70 years. The most abundant type of MV was derived from platelets, a finding verified by two different platelet surface markers. Many of these were positive for phosphatidylserine. These results provide a baseline reference range from which to: 1) design larger population studies which could evaluate MV by age, sex, menopausal status, and race in healthy controls and patient populations; 2) identify early pathophysiological processes in asymptomatic persons at risk for disease; 3) develop diagnostic, prognostic assessment, and management of individuals with suspected and established cardiovascular diseases [1-8,29].

Results of the present study indicating that numbers of phosphatidylserine, P-selectin, platelet- and endothelium-derived MV were significantly higher in apparently healthy women compared to age-matched men corroborate with previous results of populations of MV measured in platelet poor plasma by flow cytometry from young healthy women and men (32 ± 8.5 and 29 ± 3.2 years old women and men, respectively) [34]. That study also demonstrated that numbers of blood-borne MV were influenced by the phase of the menstrual cycle in premenopausal women, which could contribute to a procoagulant state in premenopausal women [34]. The present study also showed that premenopausal women had significantly higher phosphatidylserine, P-selectin positive, and MV from endothelium, although the stage of menstrual cycle was not collected in the present study. In addition, MV from erythrocytes and stem/progenitor cells were lower in premenopausal women than age-matched men. Since most of these differences were not observed in postmenopausal women compared to age-matched men, it is likely that the differences are mediated in part by changes in sex steroid hormones with menopause and aging. However, future studies will be required to evaluate the contribution of sex hormonal status and MV distribution in women and men as they age.

The circulating stem/progenitor cells and their MV in the blood contribute in various cardiovascular pathophysiological processes [35-39]. The number of circulating endothelial progenitor cells were significantly lower in coronary artery disease patients compared to healthy controls and predicts future cardiovascular events [36]. Number of senescent cells increase with aging in different parts of the body and also contribute in cardiac disease [40,41]. However, neither of these types of MV correlated with age in the present study which may reflect the general health of the participants.

Erythrocyte-derived MV increased with age in women. Erythrocytes shed MV throughout their life span to remove unwanted aged molecules from the cytoplasm and cell membrane to maintain erythrocyte function [42,43]. In male rats, erythrocyte-derived vesicles were rapidly cleared from the circulation by liver Kupffer cells through a scavenger receptor [44]. The present study showed that MV from erythrocytes were significantly higher in postmenopausal compared to premenopausal women. The increased erythrocyte-derived MV with age in women may be due either to increased MV generation or decreased clearance with aging. However, the exact clearance mechanisms and half-life of blood-borne MV in healthy humans are not yet established due to ethical considerations associated with the use of various labeling, imaging, and sampling techniques needed for such determinations.

In men, adipocyte-derived MV increased with age. Extracellular vesicles (exosomes/microvesicles) released from dysfunctional and hypertrophic adipocytes impair the function of vascular endothelium [45]. In male mice, primary adipocyte-derived extracellular vesicles stimulated angiogenesis [46]. Expansion of visceral adipose tissue due to local inflammation-mediated adipocyte hypertrophy, infiltration of inflammatory macrophages, and subsequent dysfunction of adipocytes is an independent risk factor for cardiovascular morbidity and mortality [47-50]. The increased adipocyte-derived MV in men with age may reflect sex differences in central abdominal adiposity and its association with cardiovascular disease [51]. Numbers of monocyte- and endothelium-derived MV correlated with waist circumference in recently menopausal women [21]. Unfortunately, adipocyte-derived MV were not evaluated in that study, and the relationship between these MV and waist circumference reflecting abdominal obesity in women remains to be determined.

## Conclusions

In apparently healthy blood donors, sex- and age-specific differences in total procoagulant, platelet-, endothelium-, erythrocyte-, and adipocyte-derived MV may partly contribute to differences in the incidence and progression of cardiovascular disease between women and men. Sex differences in total procoagulant and specific cell-derived populations of blood-borne MV may reflect hormonal regulation with menopausal status or compensatory mechanisms to maintain normal hemostasis associated with blood loss during menstruation in premenopausal women. Further research is needed to determine how these sex differences in blood-borne MV are associated with hormonal status in women and the relationship between adipocyte-derived MV with abdominal obesity (waist circumference) in men and women. These results provide a bench mark of ranges for various cell-derived blood-borne MV >0.2 μm in women and men from 20–70 years old from which future studies can be designed to validate the reference ranges for blood MV by age, sex, hormonal status, and race in various patient populations or asymptomatic individuals at risk for cardiovascular, thrombotic, or other diseases.

## Abbreviations

CD: cluster of differentiation
ICAM-1: inter cellular adhesion molecule-1
MV: microvesicles
VCAM-1: vascular cell adhesion molecule-1
